# Editorial

**DOI:** 10.1120/jacmp.v5i1.1988

**Published:** 2004-05-25

This issue represents a new beginning for the JACMP— how fitting that it comes just as spring is in full bloom and all things are new. And it comes as a result of a lot of hard work from a dedicated few, a fortuitous historical event in open source publishing, and the patient and kind cooperation of our new publishing partner, Multimed Inc. Has the transition gone as expected? No, it was about four times more difficult than expected. This is mostly due to the inexperience of your humble servant. However, reflecting my irrational optimism, I believe the next issue will be easier and the one after that easier still.

I would like to thank the many who brought us this far. Many thanks to Chris Marshall, John Boone, and Colin Orton of the AAPM and to Ned Sternick, Jim Smathers, and Edwin McCullough of the ACMP. These men labored hard to find a workable solution to keep the American Institute of Physics as publisher while permitting the AAPM to be the owner of the JACMP. The possibility of publishing the JACMP as a supplement to *Medical Physics* was both attractive and intriguing; however, at the end of the day, the complexity and political difficulties of this potential solution proved quite difficult to solve. I also want to offer special thanks and appreciation to Edwin McCullough, Editor‐in‐Chief for 2003, who produced the final issue of 2003 in a sea of uncertainty and controversy.

Thanks as well to Ed Nickoloff and James Astarita. Without them, the financial solution and new cost model for the JACMP would not have become a reality. Thanks to the Public Knowledge Project director, John Willinsky. Not only is the PKP manuscript submission and exchange software free, but it is also powerful and flexible. The August 2003 release of the PKP Open Journal Systems was critical for the JACMP because it enabled the new financial model to work.

Multimed has thus far exceeded all expectations as a reliable and diligent publishing partner, and deserves our appreciation and thanks.

Thanks to John Horton, ACMP Immediate Past Chairman and JACMP Associate Editor, and to the ACMP Board of Chancellors, who agreed to continue supporting the JACMP in spite of the economic challenges the ACMP has faced over the past four years. Finally, thanks to our long‐suffering Board of Editors, who stayed the course in the face of mounting uncertainty. Their job is truly thankless, not the least because I am constantly reminding them of yet another task to do.

A new beginning also looks to the future. We have now adopted a double‐blind model for manuscript review. For future JACMP manuscript submissions the reviewers will not know the identities of the authors. University promotion and tenure review committees may feel that such a model protects the review process and legitimizes the publications of authors who are also associate editors of the Journal. In addition, we are an open source journal and part of the expanding worldwide open source community. This means many things, but most importantly it means this: The copyright of your intellectual property remains with you, the author, beginning with this issue. The JACMP is your archive of record, meaning that future publications should reference this site. However, you are free to publish or distribute this work as you see fit because you remain the owner of your manuscript and its copyright.

I will have much to say about the meaning and mission of the JACMP in future editorials. For now, it is an honor to be at this place and time and to be performing this service for the worldwide medical physics community.

Michael D. Mills

Editor‐in‐Chief

JACMP

**Table:** The Journal of Applied Clinical Medical Physics Editorial Board 2004

Editorial Board 2004	
**Editor‐in‐Chief**	Michael D. Mills
**Deputy Editor‐in‐Chief**	Timothy D. Solberg
**Associate Editor‐at‐Large**	Richard Stark
**Associate Editors**	
Radiation Oncology Physics	Nzhde Agazaryan, B. Gino Fallone
	John Gibbons, Michael Herman
	Edwin McCullough, Sasa Mutic
	Timothy Paul, Matthew Podgorsak
	Mark Rivard
Medical Imaging	Walter Huda, William Pavlicek
Radiation Measurements	Larry DeWerd
Radiation Protection & Regulations	James Deye
Nonionizing Topics	Bhudatt Paliwal
Other Topics	Timothy Solberg
Book/Media Reviews	James Smathers
Editorials	John Horton
**Journal Production Team**	
Layout Editor and Proofreader	Tracy Skelton
Copy Editor	Betty R. Robinson
Manuscript Manager	Patricia Walker

